# Structure-Guided Tooth Numbering and Lesion Localization in Visible Light Oral Images

**DOI:** 10.3390/jimaging12060256

**Published:** 2026-06-09

**Authors:** Yuhuang Lin, Youcheng Luo, Fengzhen Gao, Quanjian Dong, Xinqun Lei, Bin Huang, Yendo Hu

**Affiliations:** 1 School of Computer Science and Engineering, Jimei University, Xiamen 361021, China; dust@jmu.edu.cn (Y.L.); 202221331017@jmu.edu.cn (Y.L.); 202311835002@jmu.edu.cn (F.G.); 202221331083@jmu.edu.cn (Q.D.); 202221331085@jmu.edu.cn (X.L.);; 2 School of Ocean Information Engineering, Jimei University, Xiamen 361021, China

**Keywords:** tooth numbering, oral lesion detection, visible light imaging

## Abstract

This study presents a structure-aware inference framework for tooth numbering and lesion localization in visible light oral images. Tooth numbering is often compromised by class imbalance and structural inconsistency caused by the uneven distribution of tooth types, motivating the integration of anatomical priors into the inference process. The framework first partitions the dental arch into quadrants using a deep learning-based detection module to establish spatial organization. Based on this, an Anchor-Teeth-Guided Inference (ATGI) strategy reconstructs globally consistent tooth numbering by leveraging dental arch continuity, bilateral symmetry, and confidence-guided anchor selection, thereby improving the recognition of underrepresented tooth classes. Visually suspicious lesion regions are independently detected and spatially associated with numbered teeth, enabling joint structural and lesion-aware analysis. Evaluated on a multi-source dataset, the method achieves a weighted F1-score of 0.813 for 32-class tooth numbering, outperforming end-to-end baselines while improving spatial consistency. Lesion localization yields F1-scores of 0.850 for caries-related regions and 0.789 for gingivitis-related regions. These results demonstrate that incorporating anatomical constraints enhances numbering robustness and improves rare-class recognition in visible light dental image analysis, showing potential for screening-oriented oral assessment and teledentistry applications.

## 1. Introduction

In recent years, the widespread adoption of artificial intelligence (AI) in medical image analysis has accelerated the development of intelligent image analysis and computer-assisted assessment systems. Within the field of dental care, AI is increasingly being applied to oral screening, tooth numbering, and treatment planning assistance, showing growing potential in teledentistry applications [1]. This trend is particularly relevant as aging populations and the prevalence of chronic oral diseases continue to rise, increasing the demand for efficient, accessible, and low-barrier oral health assessment tools. Such technologies may help improve the efficiency of preliminary oral screening and support broader public health initiatives.

Currently, most intelligent dental image analysis systems are based on X-ray modalities [2], which offer strong tissue penetration and high contrast. End-to-end convolutional neural networks (CNNs) have achieved promising results in unified disease classification and tooth detection tasks on X-ray images [3,4,5,6], demonstrating high accuracy and consistency on standardized datasets. However, such approaches typically rely on professional equipment and standardized acquisition protocols, making them unsuitable for home-based or telemedicine scenarios. Furthermore, the inherent radiation risk of X-ray imaging—especially for pregnant women and children—limits its application in low-barrier, routine monitoring solutions.

In contrast, visible light imaging presents a non-invasive, cost-effective, and easy-to-capture alternative. Recent studies have suggested that photographic oral screening may provide practical value for preliminary oral assessment and remote triage, particularly in low-resource or home-use settings [7]. In addition, certain oral conditions, such as caries-related discoloration and gingival inflammation, may exhibit visually identifiable features in visible light images [8,9], supporting the feasibility of image-based screening approaches.

Despite these advantages, research on visible light-based dental analysis remains limited. Some studies have explored the use of CNNs for detecting dental diseases or tooth regions in visible light images [10,11], but most are restricted to image-level classification tasks and lack the capacity to model structural relationships or provide interpretable localization. Challenges such as variable lighting, occlusions, and non-uniform samples often degrade the performance of end-to-end models, leading to low robustness and high false detection rates in tooth numbering and lesion localization tasks. Existing methods largely overlook the spatial distribution of teeth within anatomical structures and fail to incorporate priors that guide inference and associate diseases with specific teeth, making joint modeling both fragile and opaque.

To address these challenges, this paper focuses on tooth numbering and lesion localization in visible light oral images under home-use scenarios and proposes a structure-aware analysis framework that integrates a cascaded detection strategy with prior-driven inference. Specifically, the proposed framework leverages the anatomical order and bilateral symmetry of teeth and is composed of three main stages: (1) jaw detection using object detectors to localize the upper and lower dentitions, enabling instance-level segmentation and rough positioning; (2) anchor teeth classification followed by Anchor-Teeth-Guided Inference (ATGI), which infers complete tooth positions based on spatial priors and symmetry; and (3) lesion detection and semantic alignment, where a disease detector identifies caries or gingivitis regions and associates them with numbered tooth instances, thus fusing structural and pathological information.

Evaluated on a multi-source visible light dataset collected under diverse home-use conditions, our method demonstrates superior robustness and generalization. The numbering module achieved a weighted F1-score of 0.813, significantly outperforming a baseline end-to-end model (F1 = 0.687), highlighting the effectiveness of structure-aware reasoning.

In summary, this paper makes the following contributions:

- We propose a structure-aware tooth enumeration strategy that incorporates anatomical priors to improve numbering consistency and enhance the recognition of underrepresented tooth classes;
- We develop a modular framework for joint tooth numbering and lesion-aware localization with FDI-based spatial mapping, enabling interpretable association between visually suspicious regions and specific teeth;
- We construct and annotate a multi-source visible light oral image dataset to support research on tooth numbering and lesion localization in non-radiographic dental imaging scenarios.

### Related Work

In the field of dental disease detection, Chen I. D. S. [12] applied YOLOv7 [13] for single-tooth detection by automatically cropping individual tooth images from periapical radiographs.

They combined this with Contrast-Limited Adaptive Histogram Equalization (CLAHE) and bilateral filtering for image enhancement. A multi-label classification model was then used to simultaneously identify periodontitis and dental caries.

George et al. [14] trained a YOLOv8 object detection model to recognize caries, periodontitis, and oral cancer from panoramic dental X-rays. Li et al. [15] conducted a study on the auxiliary diagnosis of periodontal diseases using bitewing radiographs. They applied Gaussian filtering and adaptive binarization for preprocessing, followed by YOLOv4 [16] for tooth localization and auto-cropping. Further enhancement methods, such as contrast improvement, were used to improve lesion visibility, and a CNN-based model was employed to classify caries, restorations, and periodontal conditions.

For tooth detection and numbering in bitewing radiographs, a study [17] utilized the Mask R-CNN [18] model to achieve high-precision instance segmentation of teeth and applied the FDI World Dental Federation notation system for tooth numbering. Another study [19] employed YOLOv5, a deep learning model, for the automatic detection, segmentation, and numbering of mixed dentition in pediatric panoramic X-rays. Tuzoff et al. [20] adopted a two-stage approach for tooth numbering in panoramic radiographs: first, Faster R-CNN [21] was used to detect teeth and generate bounding boxes; then, a VGG-16 [22] classifier was employed to predict the probability of each tooth class (32 categories). A heuristic postprocessing step ensured the uniqueness of the final numbering.

Almalki et al. [23] proposed a novel method integrating self-supervised learning with a Transformer architecture. They were the first to apply a self-supervised pretraining strategy to the Swin Transformer for dental instance segmentation and tooth numbering.

## 2. Materials and Methods

### 2.1. Materials

In the field of tooth detection and numbering, accurate identification and annotation of individual teeth are important for dental image analysis, treatment planning support, and electronic medical record documentation.

To enable standardized annotation and efficient information exchange, the dental community commonly employs the Tooth Numbering System (TNS) to assign a unique identifier to each tooth. Among these systems, the two-digit numbering scheme proposed by the Fédération Dentaire Internationale (FDI), known as the FDI notation, is widely adopted and has been standardized as ISO 3950 by the International Organization for Standardization [24]. This system encodes the position of each tooth using a two-digit number: the first digit denotes the quadrant (1–4, corresponding to the upper right, upper left, lower left, and lower right, respectively), and the second digit represents the tooth’s position within that quadrant (1–8, counted from the midline posteriorly).

In this study, we adopt the FDI notation as the unified standard for tooth numbering to ensure the generalizability and interpretability of model outputs.

**Figure 1:**
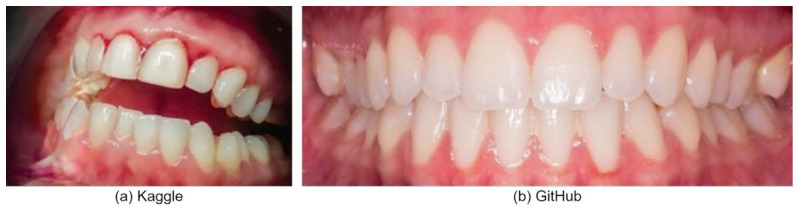
Examples from different dataset sources. (**a**) Kaggle, (**b**) GitHub.

We constructed a comprehensive dataset of 845 visible light oral cavity images for the tasks of automatic tooth numbering and lesion detection. The images are sourced from two origins (Figure 1):

- Public datasets from Kaggle [25]: The dataset includes five types of dental diseases, such as caries and gingivitis. We selected a subset of high-quality images focusing on caries and gingivitis for our experiments;
- Open-source dental image repositories on GitHub [26]: Most of the images are front or side views of relatively clean and well-aligned teeth, suitable for training structural detection models;

The dataset contains high-resolution frontal and lateral oral photographs, suitable for downstream analysis. We split the dataset into a training set (676 images) and a test set (169 images) at an 80:20 ratio.

The image annotations were conducted by three trained annotators who received guidance from in-service dental doctors regarding visually observable oral conditions and tooth numbering principles. The annotation process consisted of two components: (1) tooth numbering annotation and (2) lesion annotation.

For tooth numbering, each tooth instance was annotated with a bounding box and assigned a corresponding FDI-based identifier following standard tooth indexing rules. For lesion annotation, regions of pathological conditions were labeled based on clearly identifiable visual features in intraoral photographs, including enamel discoloration, cavitation-like regions, loss of tooth structure, gingival redness, and soft tissue swelling. These visual criteria were used as the primary basis for consistent labeling across annotators. All annotations were ultimately reviewed and verified by in-service dental doctors. Although multiple annotators followed the same annotation guidelines and underwent clinician-guided training, no formal inter-annotator agreement analysis (e.g., Cohen’s kappa or similar quantitative measures) was performed. Therefore, the consistency of the annotations was ensured through expert review rather than quantitatively assessed agreement metrics.

To facilitate model design and class balance analysis in the tooth numbering task, we categorized and analyzed the dataset based on: upper vs. lower jaw, left vs. right side and tooth position index within quadrants (1–8).

**Figure 2:**
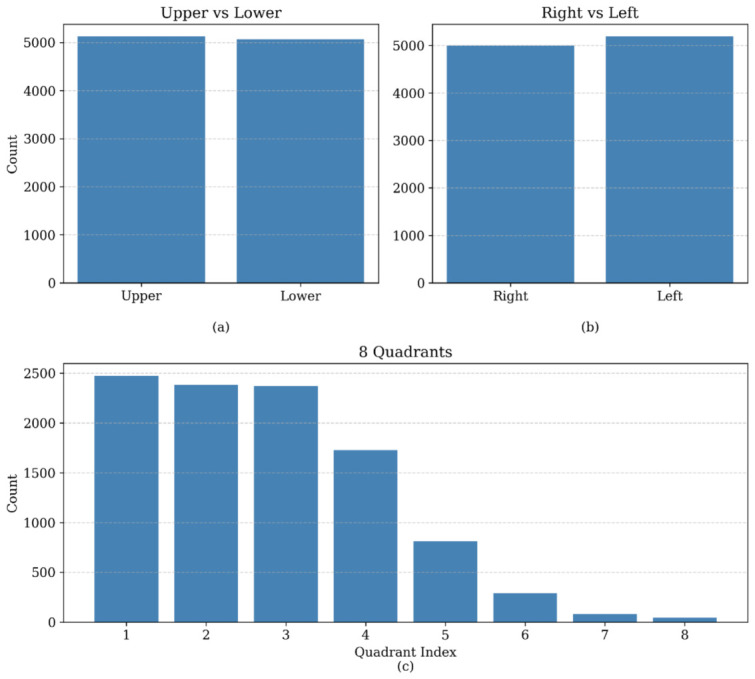
Sample distribution under three classification schemes. (**a**) Distribution of samples between upper and lower dentitions; (**b**) distribution of samples between left and right dentitions; and (**c**) distribution of samples across tooth indices (1–8) within each quadrant.

As shown in Figure 2, the data distribution is relatively balanced across upper/lower and left/right divisions, with more than 5000 samples each. However, within each quadrant, significant class imbalance exists among different tooth indices, particularly for teeth 5–8, where the sample counts are notably lower. Such imbalance may limit the ability of direct detection or classification models to learn representative features for low-sample tooth categories, resulting in structurally inconsistent numbering predictions and reduced recognition performance for posterior teeth. Since posterior teeth are clinically important regions with a high prevalence of caries-related lesions, insufficient sensitivity in these categories may affect the reliability of screening-oriented dental image analysis. This challenge further motivates the introduction of structure-aware inference to improve robustness for underrepresented tooth classes.

### 2.2. Methodology

#### 2.2.1. Overall Framework and Strategy Overview

This study proposes a modular and structure-aware framework for tooth numbering and lesion localization in visible light oral images. Unlike conventional end-to-end tooth numbering approaches that directly predict tooth identities from local image features, the proposed framework decomposes the task into structural perception, anchor-based numbering inference, and lesion–tooth association, thereby enabling anatomically consistent reasoning under class imbalance and spatial ambiguity.

The core contribution of the framework is the proposed Anchor-Teeth-Guided Inference (ATGI) strategy, which reconstructs globally consistent tooth numbering by leveraging dental arch continuity, bilateral symmetry, confidence-guided anchor selection, and FDI-based sequential constraints. Rather than treating tooth numbering as an independent multi-class classification problem, the proposed method formulates it as a structure-constrained inference process over spatially ordered tooth sequences. This hierarchical decomposition improves class separability, reduces direct classification ambiguity, and introduces an explicit intermediate semantic layer for logical correction and global consistency reconstruction.

In the system architecture:

- A YOLO-based detection module is employed for tooth instance localization and upper/lower jaw separation by embedding jaw labels into detection categories;
- An intra-quadrant tooth classifier provides preliminary positional priors for subsequent structural inference;
- The ATGI module identifies high-confidence and spatially consistent tooth subsequences as structural anchors and infers uncertain tooth numbers through FDI-guided sequence reconstruction;
- An independently trained lesion detector identifies visually suspicious pathological regions, which are subsequently associated with numbered teeth through spatial mapping.

Importantly, YOLO-based models in this framework serve only as modular perception components, while the primary methodological contribution lies in the structure-driven inference and reconstruction mechanism. This decoupled design enhances adaptability to non-structured pathological features while maintaining structural consistency between anatomical and lesion information.

As a result, the proposed framework forms a complete tooth structure and lesion-aware analysis pipeline for screening-oriented dental image assessment.

#### 2.2.2. Tooth Detection and Upper/Lower Jaw Separation

Before completing the overall structural perception of teeth, it is essential to accurately detect individual tooth instances in the images and perform structural separation of the upper and lower jaws based on the detection results. To achieve this, the study employs the YOLOv8n model as the object detection module for the automatic identification and classification of tooth regions in the original images.

During detection, the system first applies Non-Maximum Suppression (NMS) to the model’s output to eliminate redundant candidate boxes. An initial NMS threshold of 0.4 is set to retain boxes with lower mutual overlap (IoU) while favoring higher confidence scores. Subsequently, all detected boxes undergo a confidence filtering step, where detections with confidence scores below 0.3 are discarded, thereby improving the final result’s reliability and robustness.

The YOLOv8n model is trained as a binary classifier, with class labels encoded as 0 and 1, corresponding to Upper Jaw Teeth (Upper) and Lower Jaw Teeth (Lower), respectively. For each detected tooth instance $T_{i}$, if its predicted class label $c_{i}=0$, it is added to the upper jaw set U; if $c_{i}=1$, it is added to the lower jaw set L. Formally, this can be expressed as:

$$U\leftarrow U\cup \{T_{i}\},\;\mathrm{if}\;c_{i}=0$$

$$L\leftarrow L\cup \{T_{i}\},\;\mathrm{if}\;c_{i}=1$$

where $c_{i}$ denotes the class prediction of tooth $T_{i}$.

To further normalize the spatial ordering of teeth, the system sorts the detected teeth within each jaw group based on the horizontal coordinate of their bounding box centers (*x*-axis position). This ensures spatial consistency from left to right within the same jaw, establishing a sequential basis for subsequent quadrant division and tooth number inference.

Through the above procedures, the system achieves robust tooth detection and structured upper/lower jaw separation, thereby establishing the essential spatial framework required for the tooth numbering task.

#### 2.2.3. Anchor-Teeth-Guided Inference Algorithm

After completing the detection and structural separation of upper and lower jaw teeth, the system further needs to achieve precise numbering for each individual tooth. In the initial design, the study employs the YOLOv8n-cls model to perform quadrant-based numbering classification on the separated upper and lower jaw tooth lists, predicting each tooth as one of the classes from 1 to 8. Although this method can quickly provide preliminary numbering, it often suffers from structural inconsistencies such as numbering jumps, duplicates, and left–right mirror errors caused by local occlusions in tooth arrangements and classification confusion among adjacent categories.

To address these issues, this study proposes an anchor-teeth-guided inference algorithm, which integrates spatial ordering and numbering patterns to automatically extract high-confidence and rule-consistent subsequences from the detection results as anchor points and then infer the numbering of remaining teeth bidirectionally based on these anchors, thus structurally correcting the initial classification outputs.

According to the standard FDI numbering rule, teeth in a single jaw are arranged from distal (8) to mesial (1), and the numbering follows the bilateral pattern:

$${\mathrm{Rule}}_{FDI}=\left[8,7,6,5,4,3,2,1,1,2,3,4,5,6,7,8\right]$$

**Figure 3:**
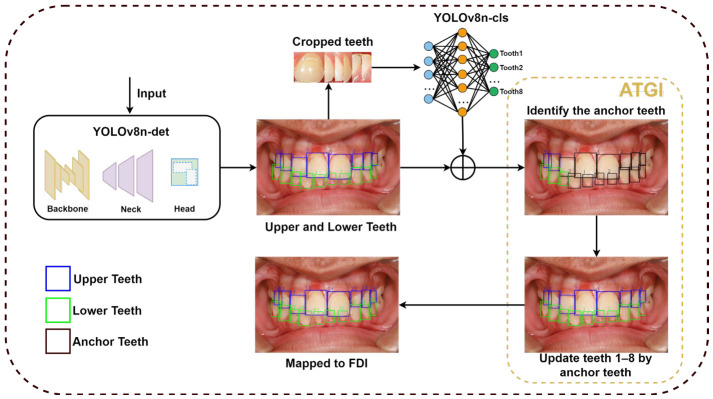
Illustration of the main process of tooth numbering.

The system searches the sorted upper and lower jaw tooth lists for the longest subsequence of predicted numbers matching this pattern. If this subsequence is spatially continuous and exhibits a monotonic numbering trend, it is designated as the anchor teeth (highlighted by black boxes in Figure 3). The structure and spatial positions of these anchor teeth serve as the foundation for subsequent numbering inference.

Once extracted, the system extends numbering to adjacent teeth on both sides based on spatial order and numbering trend, completing the numbering of teeth instances inaccurately classified by the initial model. For each unnumbered tooth, if it lies to the left (right) of the anchor teeth, the number is inferred by incrementing (decrementing) from the anchor numbering and proceeding until all teeth are assigned. To prevent error propagation into false positives or abnormal areas, maximum inference intervals and confidence thresholds are set.

The core Algorithm 1 workflow is summarized as follows:
**Algorithm 1:** Anchor-Teeth Guided Inference Algorithm.**Input:** Sorted predicted teeth list *P* with class labels and probabilities **Output:** Refined teeth list with corrected class labels
Define reference rule:R = [8,7,6,5,4,3,2,1,1,2,3,4,5,6,7,8]Extract predicted labels Lp and probabilities Sp from PInitialize:max_len = 0best_pred_start = −1best_ref_start = −1best_score = −1For each r in [0, |R|):For each p in [0, |Lp|):
⚬match_len = 0, score = 0⚬While R[r + match_len] == Lp[p + match_len]:
▪score += Sp[p + match_len]▪match_len += 1⚬If (match_len > max_len) OR(match_len == max_len AND score > best_score):
▪Update best_pred_start, best_ref_start▪max_len = match_len▪best_score = scoreIf max_len > 0For each index i in P:
⚬ref_idx = best_ref_start + (i − best_pred_start)⚬If 0 ≤ ref_idx < |R|:
▪P[i].cls = R[ref_idx]Return P


The inferred tooth-number sequence is then converted back to FDI notation using predefined mappings for the upper and lower jaws. For upper jaw teeth, the left half of the sequence corresponds to the first quadrant (numbers 18–11 in reverse order), while the right half corresponds to the second quadrant (numbers 21–28 in forward order). The same mapping principle is applied to the lower jaw.

Despite the structural robustness introduced by ATGI, the proposed inference strategy still relies on partial anatomical continuity and spatial ordering assumptions within the dental arch. In cases involving severe crowding, tooth agenesis, multiple missing teeth, extensive restorations, or significant anatomical deformation, the structural sequence may become discontinuous or ambiguous, potentially affecting anchor selection and global numbering reconstruction. Although confidence-guided filtering and constrained inference help reduce error propagation, these challenging clinical scenarios remain a limitation of the current framework and require further investigation with more clinically diverse datasets.

#### 2.2.4. Lesion Detection and Tooth Position Matching Strategy

After completing the tooth numbering inference, the system further locates lesion areas within the oral images and precisely assigns them to the corresponding tooth numbers. For this purpose, an independently trained YOLOv8n detection model is employed to identify dental lesions in the images, with target classes including caries and gingivitis.

Given an input image, the model inference outputs a set of lesion bounding boxes:

$$B=\{B_{j}=\left(x_{j},y_{j},c_{j}\right){\}}_{j=1}^{n}$$

where $\left(x_{j},y_{j}\right)$ denote the center coordinates of the predicted lesion box, and $c_{j}$ represents the predicted lesion category.

The previously numbered tooth list is denoted as:

$$T=\{T_{i}=\left(x_{i},y_{i},{\mathrm{FDI}}_{i}\right){\}}_{i=1}^{m}$$

where $\left(x_{i},y_{i}\right)$ represent the center coordinates of the tooth instance, and ${\mathrm{FDI}}_{i}$ is its inferred FDI number.

For each lesion box $\left(B_{j}\in B\right)$, the system performs Euclidean distance minimization to find the closest tooth instance $T_{i}^{*}$:

$$T_{i}^{*}=\arg\underset{T_{i}\in T}{\min}|\left(x_{j},y_{j}\right)-\left(x_{i},y_{i}\right){|}_{2}$$

Upon successful matching, the lesion label $c_{j}$ detected by $B_{j}$ is assigned to the tooth number ${\mathrm{FDI}}_{i}$ of $T_{i}$, establishing a one-to-one correspondence between the lesion and tooth position.

The final output is represented as:

$$L=\{\left({\mathrm{FDI}}_{i^{*}},c_{j}\right)|B_{j}\in B\}$$

## 3. Results

To evaluate the effectiveness of the proposed structure-aware framework, we conducted a comprehensive performance analysis across key tasks: upper/lower jaw detection, tooth classification, and lesion detection. All models were evaluated using precision, recall, and F1-score. In addition to the primary YOLOv8n-based models, YOLOv12 [27] and Faster-RCNN were implemented for comparative analysis.

All YOLO-based models were trained using the SGD optimizer with an initial learning rate of 0.01, momentum of 0.937, and weight decay of 0.0005. The batch size was set to 64, and training was conducted for 300 epochs. The input resolution was 640 × 640 for detection tasks and 224 × 224 for classification tasks. Early stopping was applied with a patience of 100 epochs to reduce overfitting. No additional customized data augmentation strategies were introduced beyond the standard training pipeline. For non-YOLO baseline models, training settings were kept consistent with those of the YOLO-based models where applicable. All experiments were conducted on a single NVIDIA A16 GPU to ensure fair and consistent computational conditions.

The manuscript was prepared with reference to the CLAIM reporting recommendations to improve methodological transparency and reproducibility. However, no formal reporting checklist was completed or submitted.

Table 1 summarizes the performance of the upper/lower jaw detection task. Building upon this foundation, an 8-class classification model (YOLOv8n-cls) was employed to infer the coarse positional information of each tooth.

To specifically evaluate the contribution of structural priors, a controlled experimental setting was designed in which all test-set teeth were assumed to be correctly detected and assigned upper or lower jaw labels, while quadrant-level annotations were not provided. This setting isolates the role of the proposed inference strategy from the base detection and classification components.

Under this configuration, the Anchor-Teeth-Guided Inference (ATGI) module was introduced as a post-processing step to complete the tooth numbering sequence by exploiting dental arch symmetry and anchor-based spatial constraints.

As shown in Table 2, the baseline classification model exhibits uneven performance across tooth categories, with relatively weaker results in low-frequency classes (Classes 5–8). In contrast, the integration of the ATGI post-processing step consistently improves quadrant-level assignment accuracy across all categories, with more pronounced gains in the small-sample classes (Classes 5–8). By incorporating structural priors and dental arch symmetry constraints, ATGI effectively compensates for insufficient class-specific discriminative information, leading to significant improvements in recall and F1-score for these categories. Overall, the results demonstrate that ATGI enhances global consistency and robustness in tooth enumeration, particularly under class imbalance conditions.

**Figure 4:**
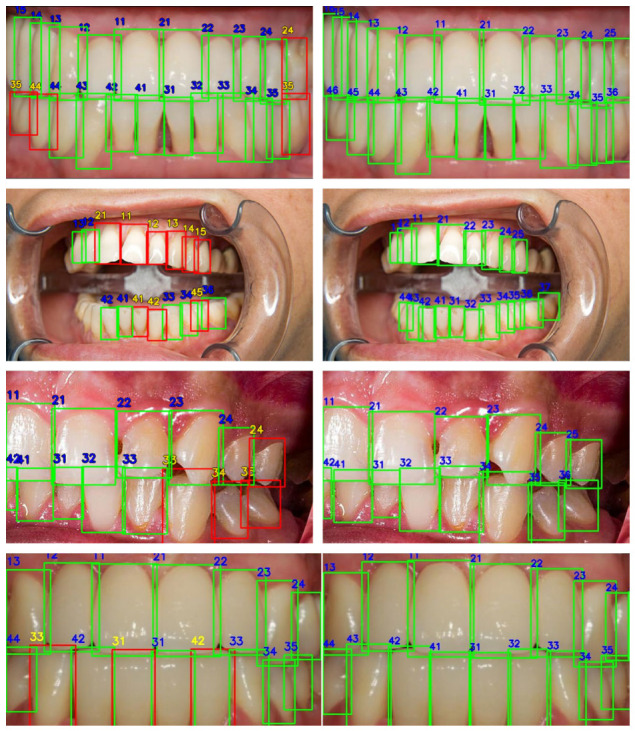
Visualization comparison between YOLOv12s (**left**) and our method (**right**), with teeth numbered according to the FDI system. Green boxes with blue text indicate correctly detected teeth with accurate FDI-based classification, while red boxes with yellow text indicate misclassified teeth.

As shown in Figure 4, the visualization comparison between YOLOv12s and our method demonstrates the effectiveness of the proposed approach. YOLOv12s is prone to classification confusion, whereas our method achieves more accurate tooth detection and FDI-based numbering.

**Figure 5:**
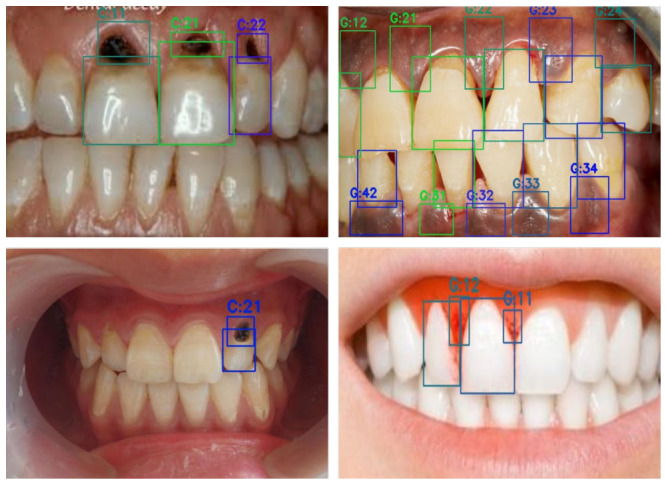
Lesion Localization Results (C: caries, G: gingivitis; the number indicates the corresponding FDI tooth numbering of each lesion instance; each lesion instance is marked with a unique color).

For lesion detection, the results are shown in Table 3. The YOLOv8n-based model achieved a precision of 0.905, recall of 0.797, and F1-score of 0.850 for caries, and a precision of 0.799, recall of 0.778, and F1-score of 0.789 for gingivitis, outperforming the YOLOv12 baseline. Visualizations (Figure 5) confirmed consistent localization accuracy and correct lesion-to-tooth mapping.

In the full 32-class tooth numbering task, the hybrid approach integrating YOLOv8n-based classification with ATGI achieved macro-averaged precision, recall, and F1-score values of 0.558, 0.582, and 0.567, respectively, with weighted averages of 0.806, 0.822, and 0.813 (Table 4). For the proposed method, values in parentheses indicate the corresponding 95% confidence intervals estimated via bootstrap resampling. The proposed approach outperformed Faster-RCNN, YOLOv8, and YOLOv12 models across both macro-averaged and weighted-averaged metrics.

## 4. Discussion

Error analysis revealed that baseline models such as YOLOv12s frequently produced repeated or anatomically inconsistent tooth numbering results, particularly under partial occlusion or uneven tooth visibility. These findings suggest that directly formulating tooth numbering as a local appearance-based multi-class classification problem may be insufficient for visible light oral images, where illumination variation, perspective distortion, and structural ambiguity are common. In contrast, the proposed Anchor-Teeth-Guided Inference (ATGI) framework introduces a structure-aware reasoning mechanism based on dental arch continuity, bilateral symmetry, and FDI-guided sequential constraints. By reconstructing globally consistent numbering from high-confidence anchor subsequences, the proposed framework improves spatial consistency and rare-class recognition under class imbalance conditions. These results indicate that incorporating anatomical priors may provide a practical alternative to purely appearance-driven tooth numbering strategies in non-radiographic dental image analysis.

From a clinical perspective, visible light oral imaging offers several advantages for screening-oriented oral assessment. Compared with radiographic imaging, visible light photography is non-invasive, radiation-free, low-cost, and easily accessible through consumer-grade devices such as smartphones. These characteristics make it potentially valuable for teledentistry, home-based oral monitoring, and preliminary triage applications, particularly in low-resource settings or populations requiring reduced radiation exposure, such as children and pregnant individuals. However, visible light photographs alone cannot provide a definitive clinical diagnosis. In routine dental practice, caries assessment often requires tactile probing, drying procedures, and radiographic examination, while gingivitis evaluation additionally depends on periodontal probing and bleeding assessment [8,9]. Therefore, the proposed framework should be interpreted as a screening-oriented structural analysis system for identifying visually suspicious regions rather than a standalone diagnostic tool. Despite these limitations, the framework demonstrates that anatomically guided structural reasoning can improve the interpretability and spatial consistency of visible light dental image analysis, which remains challenging for purely appearance-based approaches.

Another limitation lies in the dataset characteristics and clinical representativeness. The current dataset was collected from publicly available sources and mainly contains relatively clean and well-aligned oral photographs. Complex clinical conditions, including severe crowding, tooth agenesis, mixed dentition, orthodontic appliances, and extensive restorations, remain underrepresented. In addition, demographic and clinical metadata were unavailable for most images, limiting subgroup analysis across different patient populations. Furthermore, although all annotations were reviewed by in-service dental doctors, formal inter-annotator agreement metrics were not collected during dataset construction. Consequently, the reliability of the annotation process was not quantitatively assessed, which may introduce potential variability in both tooth numbering and lesion labeling. Since the proposed structure-aware inference mechanism partially relies on assumptions of anatomical continuity and spatial ordering, performance degradation may occur in cases involving missing teeth, severe decay, or disrupted dental arch structures. Future studies should therefore incorporate more clinically diverse and prospectively collected datasets, together with standardized annotation protocols and quantitative agreement assessment, to better evaluate robustness and reproducibility in realistic clinical environments.

The current lesion–tooth association strategy is based on Euclidean distance minimization between lesion centroids and tooth centers. Although computationally efficient and suitable for coarse structural association, this approach represents an approximate spatial matching strategy rather than a definitive pathological attribution method. In realistic clinical scenarios, certain lesions may not correspond to a single anatomically nearest tooth. For example, interproximal caries may involve adjacent teeth simultaneously, while gingival inflammation often extends across multiple teeth or gingival regions. Therefore, the proposed lesion–tooth mapping method should be interpreted as a pragmatic approximation for screening-oriented analysis rather than precise lesion localization. Future work may incorporate tooth contour segmentation, region-overlap analysis, or graph-based anatomical modeling to improve lesion–tooth correspondence under complex clinical conditions.

Overall, the proposed framework demonstrates the feasibility of combining structure-aware tooth numbering and lesion localization in visible light oral images. Future work will focus on improving robustness under clinically diverse conditions, integrating tooth segmentation and numbering into a unified framework, and extending lesion analysis to additional oral conditions such as dental calculus, crown or root fractures, and periapical pathologies. Prospective clinical validation and comparison with clinician assessment will also be necessary to further evaluate the practical applicability of the proposed framework in real-world dental screening scenarios.

## 5. Conclusions

This study presents a structure-aware framework for tooth numbering and lesion localization using visible light oral images captured under real-world conditions. By combining quadrant-level detection, Anchor-Teeth-Guided Inference (ATGI), and independent lesion analysis, the proposed method achieves improved accuracy, spatial consistency, and interpretability compared with conventional end-to-end approaches. The framework demonstrates reliable performance in tooth numbering and lesion localization tasks.

Importantly, the system is designed for non-radiographic visible light imaging, making it potentially applicable to screening-oriented oral assessment and teledentistry scenarios. Although performance may decrease in cases involving severe anatomical variation, missing teeth, or complex pathological conditions, the results suggest that incorporating anatomical priors and structure-aware inference can improve robustness in visible light dental image analysis. Future work will focus on improving adaptability to complex clinical scenarios, refining lesion-to-tooth association strategies, and expanding the range of detectable oral conditions.

## Figures and Tables

**Table 1:** YOLOv8n-det1 Model Performance.

Model	Class	Precision	Recall	F1-Score
YOLOv8n	Mean	0.842	0.863	0.853
	Upper Teeth	0.865	0.871	0.869
	Lower Teeth	0.819	0.855	0.837
YOLOv12n	Mean	0.768	0.796	0.781
	Upper Teeth	0.783	0.815	0.798
	Lower Teeth	0.753	0.778	0.764

**Table 2:** YOLOv8n-cls Model Performance.

Class	YOLOv8n-cls			YOLOv8n-cls (with ATGI)		
	Precision	Recall	F1-Score	Precision	Recall	F1-Score
1	0.908	0.915	0.912	0.917	0.931	0.924
2	0.875	0.877	0.876	0.887	0.899	0.893
3	0.870	0.890	0.880	0.897	0.882	0.889
4	0.834	0.836	0.835	0.881	0.859	0.870
5	0.742	0.695	0.717	0.802	0.814	0.808
6	0.730	0.613	0.667	0.766	0.787	0.777
7	0.480	0.500	0.490	0.625	0.600	0.612
8	0.400	0.444	0.421	0.556	0.500	0.526

**Table 3:** YOLOv8n-cls Model Performance.

Model	Class	Precision	Recall	F1-Score
YOLOv8n	Mean	0.852	0.787	0.820
	Caries	0.905	0.797	0.850
	Gingivitis	0.799	0.778	0.789
YOLOv12n	Mean	0.817	0.765	0.788
	Caries	0.836	0.739	0.784
	Gingivitis	0.797	0.790	0.793

**Table 4:** Comparative Experiment Results.

Model	Macro-Averaged			Weighted-Averaged		
	Precision	Recall	F1-Score	Precision	Recall	F1-Score
FR-CNN (Resnet50)	0.412	0.355	0.332	0.581	0.596	0.534
FR-CNN (VGG16)	0.355	0.402	0.365	0.546	0.674	0.597
YOLOv8n	0.394	0.424	0.400	0.597	0.618	0.602
YOLOv8s	0.470	0.447	0.441	0.661	0.669	0.657
YOLOv12n	0.432	0.446	0.432	0.664	0.686	0.666
YOLOv12s	0.448	0.470	0.454	0.669	0.713	0.687
**Ours**	**0.558** **(0.517–0.593)**	**0.582** **(0.540–0.624)**	**0.567** **(0.526–0.602)**	**0.806** **(0.765–0.843)**	**0.822** **(0.776–0.862)**	**0.813** **(0.769–0.851)**

The **bold** values indicate the best-performing results of the proposed method.

## Data Availability

The datasets analyzed in this study are openly available in Kaggle at https://www.kaggle.com/datasets/mohamedmahmoud153/dental-diseases/data (accessed on 23 March 2026), and in GitHub at https://github.com/PKNU-PR-ML-Lab/calculus/blob/main/normal.zip (accessed on 23 March 2026). The data generated during this study (including annotations) are available from the corresponding author upon reasonable request.

## References

[1] Schwendicke F., Samek W., Krois J. (2020). Artificial Intelligence in Dentistry: Chances and Challenges. J. Dent. Res..

[2] Bhat S., Birajdar G.K., Patil M.D. (2023). A comprehensive survey of deep learning algorithms and applications in dental radiograph analysis. Healthc. Anal..

[3] Silva B., Pinheiro L., Oliveira L., Pithon M. (2020). A study on tooth segmentation and numbering using end-to-end deep neural networks. Proceedings of the 33rd SIBGRAPI Conference on Graphics, Patterns and Images (SIBGRAPI), Porto de Galinhas, Brazil, 7–10 November 2020.

[4] Zhao Y., Li P., Gao C., Liu Y., Chen Q., Yang F., Meng D. (2020). TSASNet: Tooth segmentation on dental panoramic X-ray images by Two-Stage Attention Segmentation Network. Knowl.-Based Syst..

[5] Bui T.H., Hamamoto K., Paing M.P. (2022). Automated Caries Screening Using Ensemble Deep Learning on Panoramic Radiographs. Entropy.

[6] Ali M.d.A., Fujita D., Kobashi S. (2023). Teeth and prostheses detection in dental panoramic X-rays using CNN-based object detector and a priori knowledge-based algorithm. Sci. Rep..

[7] Kargozar S., Jadidfard M.P. (2024). Teledentistry accuracy for caries diagnosis: A systematic review of in-vivo studies using extra-oral photography methods. BMC Oral Health.

[8] Ismail A.I., Sohn W., Tellez M., Amaya A., Sen A., Hasson H., Pitts N.B. (2007). The International Caries Detection and Assessment System (ICDAS): An integrated system for measuring dental caries. Community Dent. Oral Epidemiol..

[9] Caton J.G., Armitage G., Berglundh T., Chapple I.L.C., Jepsen S., Kornman K.S., Mealey B.L., Papapanou P.N., Sanz M., Tonetti M.S. (2018). A new classification scheme for periodontal and peri-implant diseases and conditions—Introduction and key changes from the 1999 classification. J. Periodontol..

[10] Thanh M.T.G., Van Toan N., Ngoc V.T.N., Tra N.T., Giap C.N., Nguyen D.M. (2022). Deep Learning Application in Dental Caries Detection Using Intraoral Photos Taken by Smartphones. Appl. Sci..

[11] Tasmara F.A., Widyaningrum R., Setiawan A., Mitrayana M. (2023). Photoacoustic imaging of hidden dental caries using visible–light diode laser. J. Appl. Clin. Med. Phys..

[12] Chen I.D.S., Yang C.M., Chen M.J., Chen M.C., Weng R.M., Yeh C.H. (2023). Deep Learning-Based Recognition of Periodontitis and Dental Caries in Dental X-ray Images. Bioengineering.

[13] Wang C.Y., Bochkovskiy A., Liao H.Y.M. (2023). YOLOv7: Trainable Bag-of-Freebies Sets New State-of-the-Art for Real-Time Object Detectors. Proceedings of the 2023 IEEE/CVF Conference on Computer Vision and Pattern Recognition (CVPR), Vancouver, BC, Canada, 18–22 June 2023.

[14] George J., Hemanth T.S., Raju J., Mattapallil J.G., Naveen N. (2023). Dental Radiography Analysis and Diagnosis using YOLOv8. Proceedings of the 2023 9th International Conference on Smart Computing and Communications (ICSCC), Kochi, India, 17–19, August 2023.

[15] Li K.C., Mao Y.C., Lin M.F., Li Y.Q., Chen C.A., Chen T.Y., Abu P.A.R. (2024). Detection of Tooth Position by YOLOv4 and Various Dental Problems Based on CNN With Bitewing Radiograph. IEEE Access.

[16] Bochkovskiy A., Wang C.Y., Liao H.Y.M. (2020). YOLOv4: Optimal Speed and Accuracy of Object Detection. arXiv.

[17] Yaren Tekin B., Ozcan C., Pekince A., Yasa Y. (2022). An enhanced tooth segmentation and numbering according to FDI notation in bitewing radiographs. Comput. Biol. Med..

[18] He K., Gkioxari G., Dollar P., Girshick R. (2017). Mask R-CNN. Proceedings of the 2017 IEEE International Conference on Computer Vision (ICCV), Venice, Italy, 22–29 October 2017.

[19] Beser B., Reis T., Berber M.N., Topaloglu E., Gungor E., Kılıc M.C., Duman S., Çelik Ö., Kuran A., Bayrakdar I.S. (2024). YOLO-V5 based deep learning approach for tooth detection and segmentation on pediatric panoramic radiographs in mixed dentition. BMC Med. Imaging.

[20] Tuzoff D.V., Tuzova L.N., Bornstein M.M., Krasnov A.S., Kharchenko M.A., Nikolenko S.I., Sveshnikov M.M., Bednenko G.B. (2019). Tooth detection and numbering in panoramic radiographs using convolutional neural networks. Dentomaxillofac. Radiol..

[21] Ren S., He K., Girshick R., Sun J. (2017). Faster R-CNN: Towards Real-Time Object Detection with Region Proposal Networks. IEEE Trans. Pattern Anal. Mach. Intell..

[22] Simonyan K., Zisserman A. (2015). Very Deep Convolutional Networks for Large-Scale Image Recognition. arXiv.

[23] Almalki A., Latecki L.J. (2023). Self-Supervised Learning with Masked Image Modeling for Teeth Numbering, Detection of Dental Restorations, and Instance Segmentation in Dental Panoramic Radiographs. Proceedings of the 2023 IEEE/CVF Winter Conference on Applications of Computer Vision (WACV), Waikoloa, HI, USA, 3–7 January 2023.

[24] Al-Johany S.S. (2016). Tooth Numbering System in Saudi Arabia: Survey. Saudi Dent. J..

[25] Al-Akhal M.M. Dental Diseases Dataset. Kaggle. https://www.kaggle.com/datasets/mohamedmahmoud153/dental-diseases/data.

[26] PKNU PR-ML Lab (2024). Normal.zip—Calculus Repository. GitHub. https://github.com/PKNU-PR-ML-Lab/calculus/blob/main/normal.zip.

[27] Tian Y., Ye Q., Doermann D. (2025). YOLOv12: Attention-Centric Real-Time Object Detector. arXiv.

